# 2-(2-*p*-Tolyl­benzo[*g*]quinolin-3-yl)ethanol

**DOI:** 10.1107/S1600536811038736

**Published:** 2011-09-30

**Authors:** Nan Wu, Rongli Zhang, Yumei Wang, Xin Xu, Zhou Xu

**Affiliations:** a Department of Aviation Oil and Material, Xuzhou Airforce College, Xuzhou Jiangsu 221110, People’s Republic of China; b Deparement of Chemistry, Xuzhou Medical College, Xuzhou Jiangsu 221004, People’s Republic of China

## Abstract

In the title compound, C_22_H_19_NO, the pyridine ring and the adjacent naphthalene ring system are nearly coplanar, making a dihedral angle of 3.3 (1)°, while the pyridine and benzene rings are perpendicular to each other, with a dihedral angle of 89.9 (1)°. The crystal packing is stabilized by inter­molecular O—H⋯N hydrogen bonds and C—H⋯π inter­actions.

## Related literature

For the biological activity of quinoline derivatives, see: Faber *et al.* (1984 ▶); Johnson *et al.* (1989 ▶); Nesterova *et al.* (1995 ▶); Yamada *et al.* (1992 ▶).
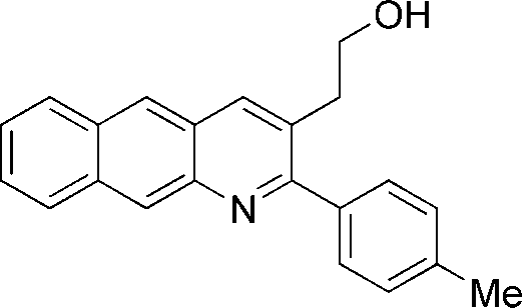


## Experimental

### 

#### Crystal data

C_22_H_19_NO
*M* *_r_* = 313.38
Triclinic, 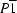

*a* = 7.2044 (4) Å
*b* = 10.1704 (4) Å
*c* = 12.1194 (3) Å
α = 108.125 (3)°
β = 98.115 (4)°
γ = 99.370 (5)°
*V* = 815.08 (6) Å^3^
*Z* = 2
Mo *K*α radiation
μ = 0.08 mm^−1^
*T* = 296 K
0.49 × 0.21 × 0.07 mm

#### Data collection

Bruker APEXII area-detector diffractometer
10164 measured reflections
2879 independent reflections
2232 reflections with *I* > 2σ(*I*)
*R*_int_ = 0.020

#### Refinement

*R*[*F* ^2^ > 2σ(*F* ^2^)] = 0.043
*wR*(*F* ^2^) = 0.127
*S* = 1.03
2879 reflections
222 parameters
H atoms treated by a mixture of independent and constrained refinement
Δρ_max_ = 0.17 e Å^−3^
Δρ_min_ = −0.16 e Å^−3^

### 

Data collection: *APEX2* (Bruker, 2004 ▶); cell refinement: *SAINT* (Bruker, 2004 ▶); data reduction: *SAINT*; program(s) used to solve structure: *SHELXS97* (Sheldrick, 2008 ▶); program(s) used to refine structure: *SHELXL97* (Sheldrick, 2008 ▶); molecular graphics: *SHELXTL* (Sheldrick, 2008 ▶); software used to prepare material for publication: *SHELXTL*.

## Supplementary Material

Crystal structure: contains datablock(s) global, I. DOI: 10.1107/S1600536811038736/hg5076sup1.cif

Structure factors: contains datablock(s) I. DOI: 10.1107/S1600536811038736/hg5076Isup2.hkl

Supplementary material file. DOI: 10.1107/S1600536811038736/hg5076Isup3.cml

Additional supplementary materials:  crystallographic information; 3D view; checkCIF report

## Figures and Tables

**Table 1:** Hydrogen-bond geometry (Å, °) *Cg* is the centroid of the N1,C1–C5 pyridine ring.

*D*—H⋯*A*	*D*—H	H⋯*A*	*D*⋯*A*	*D*—H⋯*A*
O1—H1⋯N1^i^	0.93 (3)	1.98 (3)	2.9110 (18)	174 (2)
C21—H21*A*⋯*Cg*^ii^	0.93	2.97	3.7358 (19)	140

Symmetry codes: (i) 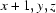; (ii) 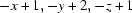.

**Fig. 1.**
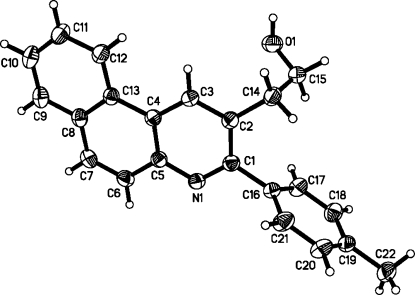
The molecular structure drawing shows 30% probability of displacement ellipsoids and the atom-numbering scheme.

**Fig. 2.**
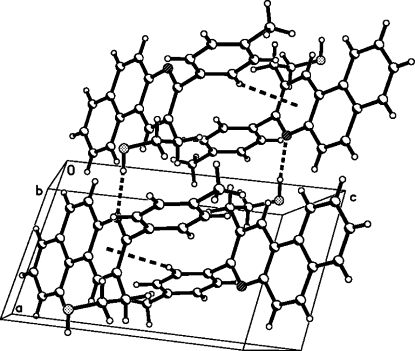
The packing diagram of title compound viewed along the b axis. Dashed lines indicate hydrogen bonds of type O1—H1···N1 and C—H···π interactions.

## supplementary crystallographic information

### Comment

Quinoline derivatives possess varies of biological properties, such as psychotropic activity (Nesterova, *et al.*, 1995), anti-allergic (Yamada *et al.*, 1992) and anti-inflammatory activity (Faber *et al.*, 1984 and Johnson *et al.*, 1989). Therefore, the title compound (Fig. 1), may be used as a new precursor for obtaining bioactive molecules. Herein, we report the crystal structure of the title compound, (I).

In the crystal structure of (I), there are four aromatic rings and the pyridine ring is the new formed ring. The pyridine ring is a coplanar conformation. The pyridine ring and the adjacent naphthalene ring are nearly coplanar, with a dihedral angle of 3.3 (1)°. While the pyridine ring and the benzene ring are vertical with each other, with a dihedral angle of 89.9 (1)°. The molecules are connected by the O1—H1···N1 intermolecular hydrogen bond and C—H···π interactions (Figure 2). Besides, there is intermolecular π-π interaction between the two neighboring benzene rings (C4C5C6C7C8C13), symmetry code: (1-*X*, 2-Y, –*Z*). The two rings are parallel to each other. The centroid distance, plane-plane distance and displacement distance are 3.642, 3.499 and 1.010 Å, respectively, which strongly indicate the existence of intermolecular π-π interactions.

### Experimental

The title compound, (I), was prepared by the reaction of 4-methylbenzaldehyde (0.240 g, 2.0 mmol), naphthalen-2-amine (0.286 g, 2.0 mmol) and I_2_ (0.051 g, 0.2 mmol) in THF (10 ml) at reflux for 40 h (yield 86%, mp. 486–487 K). Crystals of (I) suitable for X-ray diffraction were obtained by slow evaporation of a THF solution.

### Refinement

The hydrogen atom of hydroxy group, was positioned from a Fourier difference map and was refined freely. Other H atoms were placed in calculated positions, with C—H = 0.93–0.98 Å, and with *U*_iso_(H) = 1.2*U*_eq_(parent atom).

### Crystal data

**Table:**

C_22_H_19_NO	*Z* = 2
*M**_r_* = 313.38	*F*(000) = 332
Triclinic, *P*1	*D*_x_ = 1.277 Mg m^−^^3^
Hall symbol: -P 1	Melting point = 486–487 K
*a* = 7.2044 (4) Å	Mo *K*α radiation, λ = 0.71073 Å
*b* = 10.1704 (4) Å	Cell parameters from 3017 reflections
*c* = 12.1194 (3) Å	θ = 2.9–26.5°
α = 108.125 (3)°	µ = 0.08 mm^−^^1^
β = 98.115 (4)°	*T* = 296 K
γ = 99.370 (5)°	Sheet, yellow
*V* = 815.08 (6) Å^3^	0.49 × 0.21 × 0.07 mm

### Data collection

**Table:**

Bruker APEXII area-detector diffractometer	2232 reflections with *I* > 2σ(*I*)
Radiation source: fine-focus sealed tube	*R*_int_ = 0.020
graphite	θ_max_ = 25.0°, θ_min_ = 1.8°
phi and ω scans	*h* = −8→8
10164 measured reflections	*k* = −12→12
2879 independent reflections	*l* = −14→14

### Refinement

**Table:**

Refinement on *F*^2^	Primary atom site location: structure-invariant direct methods
Least-squares matrix: full	Secondary atom site location: difference Fourier map
*R*[*F*^2^ > 2σ(*F*^2^)] = 0.043	Hydrogen site location: inferred from neighbouring sites
*wR*(*F*^2^) = 0.127	H atoms treated by a mixture of independent and constrained refinement
*S* = 1.03	*w* = 1/[σ^2^(*F*_o_^2^) + (0.0628*P*)^2^ + 0.1533*P*] where *P* = (*F*_o_^2^ + 2*F*_c_^2^)/3
2879 reflections	(Δ/σ)_max_ < 0.001
222 parameters	Δρ_max_ = 0.17 e Å^−^^3^
0 restraints	Δρ_min_ = −0.16 e Å^−^^3^

### Special details

**Table:**

Geometry. All e.s.d.'s (except the e.s.d. in the dihedral angle between two l.s. planes) are estimated using the full covariance matrix. The cell e.s.d.'s are taken into account individually in the estimation of e.s.d.'s in distances, angles and torsion angles; correlations between e.s.d.'s in cell parameters are only used when they are defined by crystal symmetry. An approximate (isotropic) treatment of cell e.s.d.'s is used for estimating e.s.d.'s involving l.s. planes.
Refinement. Refinement of *F*^2^ against ALL reflections. The weighted *R*-factor *wR* and goodness of fit *S* are based on *F*^2^, conventional *R*-factors *R* are based on *F*, with *F* set to zero for negative *F*^2^. The threshold expression of *F*^2^ > σ(*F*^2^) is used only for calculating *R*-factors(gt) *etc*. and is not relevant to the choice of reflections for refinement. *R*-factors based on *F*^2^ are statistically about twice as large as those based on *F*, and *R*- factors based on ALL data will be even larger.

### Fractional atomic coordinates and isotropic or equivalent isotropic displacement parameters (Å^2^)

**Table:**

	*x*	*y*	*z*	*U*_iso_*/*U*_eq_	
O1	0.94638 (19)	0.74485 (14)	0.16847 (12)	0.0671 (4)	
N1	0.34813 (18)	0.89390 (13)	0.23730 (12)	0.0473 (3)	
C4	0.5880 (2)	1.05942 (15)	0.19621 (13)	0.0428 (4)	
C5	0.3959 (2)	0.99592 (15)	0.18921 (13)	0.0442 (4)	
C1	0.4871 (2)	0.84951 (15)	0.29047 (13)	0.0445 (4)	
C3	0.7299 (2)	1.00948 (15)	0.25305 (14)	0.0462 (4)	
H3A	0.8585	1.0487	0.2594	0.055*	
C13	0.6286 (2)	1.16905 (15)	0.14470 (13)	0.0442 (4)	
C6	0.2436 (2)	1.03711 (17)	0.12868 (15)	0.0532 (4)	
H6A	0.1167	0.9958	0.1250	0.064*	
C2	0.6844 (2)	0.90436 (15)	0.29965 (13)	0.0452 (4)	
C8	0.4736 (2)	1.20451 (15)	0.08298 (13)	0.0478 (4)	
C16	0.4202 (2)	0.73826 (16)	0.34132 (14)	0.0454 (4)	
C9	0.5119 (3)	1.30789 (17)	0.03012 (15)	0.0580 (5)	
H9A	0.4103	1.3303	−0.0115	0.070*	
C7	0.2812 (2)	1.13509 (17)	0.07688 (15)	0.0552 (4)	
H7A	0.1795	1.1583	0.0360	0.066*	
C19	0.2814 (2)	0.52892 (18)	0.43403 (17)	0.0545 (4)	
C14	0.8416 (2)	0.84739 (17)	0.35334 (15)	0.0537 (4)	
H14A	0.9579	0.9215	0.3842	0.064*	
H14B	0.8043	0.8229	0.4195	0.064*	
C18	0.3153 (2)	0.49386 (18)	0.32095 (16)	0.0594 (5)	
H18A	0.2917	0.3987	0.2741	0.071*	
C17	0.3831 (3)	0.59584 (17)	0.27504 (15)	0.0561 (4)	
H17A	0.4043	0.5683	0.1980	0.067*	
C12	0.8149 (2)	1.24194 (17)	0.15198 (16)	0.0557 (4)	
H12A	0.9188	1.2210	0.1929	0.067*	
C11	0.8479 (3)	1.34320 (18)	0.10038 (17)	0.0647 (5)	
H11A	0.9730	1.3902	0.1066	0.078*	
C15	0.8844 (2)	0.71879 (18)	0.26679 (17)	0.0583 (5)	
H15A	0.9832	0.6864	0.3079	0.070*	
H15B	0.7695	0.6433	0.2384	0.070*	
C21	0.3886 (3)	0.77368 (18)	0.45560 (16)	0.0628 (5)	
H21A	0.4142	0.8686	0.5032	0.075*	
C22	0.2070 (3)	0.4166 (2)	0.4833 (2)	0.0769 (6)	
H22A	0.2832	0.3461	0.4697	0.115*	
H22B	0.0756	0.3728	0.4446	0.115*	
H22C	0.2147	0.4591	0.5669	0.115*	
C10	0.6947 (3)	1.37576 (18)	0.03868 (16)	0.0641 (5)	
H10A	0.7174	1.4442	0.0032	0.077*	
C20	0.3197 (3)	0.6706 (2)	0.50019 (17)	0.0655 (5)	
H20A	0.2985	0.6978	0.5772	0.079*	
H1	1.074 (4)	0.793 (3)	0.196 (2)	0.111 (8)*	

### Atomic displacement parameters (Å^2^)

**Table:**

	*U*^11^	*U*^22^	*U*^33^	*U*^12^	*U*^13^	*U*^23^
O1	0.0505 (8)	0.0835 (9)	0.0729 (9)	0.0124 (6)	0.0160 (6)	0.0342 (7)
N1	0.0446 (7)	0.0461 (7)	0.0529 (8)	0.0092 (6)	0.0146 (6)	0.0176 (6)
C4	0.0459 (9)	0.0390 (7)	0.0424 (8)	0.0093 (6)	0.0116 (7)	0.0115 (6)
C5	0.0462 (9)	0.0418 (8)	0.0442 (9)	0.0108 (6)	0.0123 (7)	0.0122 (7)
C1	0.0457 (9)	0.0433 (8)	0.0445 (9)	0.0090 (7)	0.0133 (7)	0.0138 (7)
C3	0.0420 (8)	0.0455 (8)	0.0515 (9)	0.0058 (6)	0.0109 (7)	0.0185 (7)
C13	0.0518 (9)	0.0387 (8)	0.0414 (9)	0.0114 (7)	0.0109 (7)	0.0112 (6)
C6	0.0435 (9)	0.0544 (9)	0.0620 (11)	0.0125 (7)	0.0105 (8)	0.0196 (8)
C2	0.0467 (9)	0.0437 (8)	0.0458 (9)	0.0087 (7)	0.0107 (7)	0.0161 (7)
C8	0.0593 (10)	0.0411 (8)	0.0414 (9)	0.0149 (7)	0.0098 (7)	0.0101 (7)
C16	0.0404 (8)	0.0477 (8)	0.0503 (9)	0.0072 (6)	0.0123 (7)	0.0199 (7)
C9	0.0745 (12)	0.0502 (9)	0.0514 (10)	0.0205 (9)	0.0074 (9)	0.0196 (8)
C7	0.0532 (10)	0.0549 (9)	0.0581 (10)	0.0199 (8)	0.0059 (8)	0.0184 (8)
C19	0.0383 (8)	0.0633 (10)	0.0714 (12)	0.0083 (7)	0.0124 (8)	0.0373 (9)
C14	0.0480 (9)	0.0580 (10)	0.0600 (11)	0.0053 (7)	0.0062 (8)	0.0322 (8)
C18	0.0601 (11)	0.0470 (9)	0.0667 (12)	0.0011 (8)	0.0105 (9)	0.0200 (8)
C17	0.0647 (11)	0.0514 (9)	0.0492 (10)	0.0049 (8)	0.0133 (8)	0.0162 (8)
C12	0.0542 (10)	0.0522 (9)	0.0649 (11)	0.0081 (7)	0.0108 (8)	0.0283 (8)
C11	0.0668 (12)	0.0573 (10)	0.0725 (12)	0.0023 (9)	0.0126 (10)	0.0322 (9)
C15	0.0444 (9)	0.0620 (10)	0.0790 (12)	0.0120 (8)	0.0151 (9)	0.0373 (9)
C21	0.0784 (12)	0.0499 (9)	0.0585 (11)	0.0069 (8)	0.0286 (9)	0.0136 (8)
C22	0.0576 (11)	0.0869 (14)	0.1071 (17)	0.0101 (10)	0.0221 (11)	0.0628 (13)
C10	0.0861 (14)	0.0502 (9)	0.0610 (11)	0.0119 (9)	0.0129 (10)	0.0284 (9)
C20	0.0731 (12)	0.0716 (12)	0.0567 (11)	0.0087 (9)	0.0272 (9)	0.0264 (9)

### Geometric parameters (Å, °)

**Table:**

O1—C15	1.413 (2)	C7—H7A	0.9300
O1—H1	0.93 (3)	C19—C18	1.372 (3)
N1—C1	1.3294 (19)	C19—C20	1.373 (3)
N1—C5	1.3606 (19)	C19—C22	1.505 (2)
C4—C3	1.402 (2)	C14—C15	1.508 (2)
C4—C5	1.407 (2)	C14—H14A	0.9700
C4—C13	1.448 (2)	C14—H14B	0.9700
C5—C6	1.426 (2)	C18—C17	1.377 (2)
C1—C2	1.415 (2)	C18—H18A	0.9300
C1—C16	1.494 (2)	C17—H17A	0.9300
C3—C2	1.372 (2)	C12—C11	1.368 (2)
C3—H3A	0.9300	C12—H12A	0.9300
C13—C12	1.400 (2)	C11—C10	1.388 (3)
C13—C8	1.414 (2)	C11—H11A	0.9300
C6—C7	1.345 (2)	C15—H15A	0.9700
C6—H6A	0.9300	C15—H15B	0.9700
C2—C14	1.511 (2)	C21—C20	1.377 (2)
C8—C9	1.402 (2)	C21—H21A	0.9300
C8—C7	1.430 (2)	C22—H22A	0.9600
C16—C17	1.380 (2)	C22—H22B	0.9600
C16—C21	1.380 (2)	C22—H22C	0.9600
C9—C10	1.357 (3)	C10—H10A	0.9300
C9—H9A	0.9300	C20—H20A	0.9300
			
C15—O1—H1	105.8 (15)	C15—C14—H14A	108.9
C1—N1—C5	119.18 (13)	C2—C14—H14A	108.9
C3—C4—C5	116.48 (14)	C15—C14—H14B	108.9
C3—C4—C13	123.98 (14)	C2—C14—H14B	108.9
C5—C4—C13	119.54 (14)	H14A—C14—H14B	107.7
N1—C5—C4	122.42 (14)	C19—C18—C17	121.72 (16)
N1—C5—C6	117.78 (14)	C19—C18—H18A	119.1
C4—C5—C6	119.79 (14)	C17—C18—H18A	119.1
N1—C1—C2	122.65 (14)	C18—C17—C16	121.16 (16)
N1—C1—C16	115.17 (13)	C18—C17—H17A	119.4
C2—C1—C16	122.17 (14)	C16—C17—H17A	119.4
C2—C3—C4	121.90 (14)	C11—C12—C13	121.65 (17)
C2—C3—H3A	119.1	C11—C12—H12A	119.2
C4—C3—H3A	119.0	C13—C12—H12A	119.2
C12—C13—C8	117.79 (14)	C12—C11—C10	120.04 (18)
C12—C13—C4	123.31 (14)	C12—C11—H11A	120.0
C8—C13—C4	118.90 (14)	C10—C11—H11A	120.0
C7—C6—C5	120.72 (15)	O1—C15—C14	113.27 (14)
C7—C6—H6A	119.6	O1—C15—H15A	108.9
C5—C6—H6A	119.6	C14—C15—H15A	108.9
C3—C2—C1	117.33 (14)	O1—C15—H15B	108.9
C3—C2—C14	120.10 (14)	C14—C15—H15B	108.9
C1—C2—C14	122.52 (13)	H15A—C15—H15B	107.7
C9—C8—C13	119.27 (15)	C20—C21—C16	121.03 (16)
C9—C8—C7	121.50 (15)	C20—C21—H21A	119.5
C13—C8—C7	119.23 (14)	C16—C21—H21A	119.5
C17—C16—C21	117.21 (15)	C19—C22—H22A	109.5
C17—C16—C1	121.38 (14)	C19—C22—H22B	109.5
C21—C16—C1	121.40 (14)	H22A—C22—H22B	109.5
C10—C9—C8	121.18 (16)	C19—C22—H22C	109.5
C10—C9—H9A	119.4	H22A—C22—H22C	109.5
C8—C9—H9A	119.4	H22B—C22—H22C	109.5
C6—C7—C8	121.74 (15)	C9—C10—C11	120.07 (16)
C6—C7—H7A	119.1	C9—C10—H10A	120.0
C8—C7—H7A	119.1	C11—C10—H10A	120.0
C18—C19—C20	117.08 (15)	C19—C20—C21	121.80 (17)
C18—C19—C22	121.27 (17)	C19—C20—H20A	119.1
C20—C19—C22	121.65 (17)	C21—C20—H20A	119.1
C15—C14—C2	113.54 (14)		
			
C1—N1—C5—C4	−1.7 (2)	C2—C1—C16—C17	−90.9 (2)
C1—N1—C5—C6	177.53 (13)	N1—C1—C16—C21	−88.66 (19)
C3—C4—C5—N1	1.6 (2)	C2—C1—C16—C21	90.7 (2)
C13—C4—C5—N1	−179.01 (13)	C13—C8—C9—C10	−1.0 (2)
C3—C4—C5—C6	−177.56 (13)	C7—C8—C9—C10	178.41 (15)
C13—C4—C5—C6	1.8 (2)	C5—C6—C7—C8	−1.8 (3)
C5—N1—C1—C2	0.2 (2)	C9—C8—C7—C6	−179.16 (15)
C5—N1—C1—C16	179.51 (12)	C13—C8—C7—C6	0.2 (2)
C5—C4—C3—C2	−0.1 (2)	C3—C2—C14—C15	−92.86 (18)
C13—C4—C3—C2	−179.40 (13)	C1—C2—C14—C15	84.48 (19)
C3—C4—C13—C12	−3.5 (2)	C20—C19—C18—C17	−0.5 (3)
C5—C4—C13—C12	177.21 (14)	C22—C19—C18—C17	179.92 (16)
C3—C4—C13—C8	176.03 (13)	C19—C18—C17—C16	0.1 (3)
C5—C4—C13—C8	−3.3 (2)	C21—C16—C17—C18	0.7 (3)
N1—C5—C6—C7	−178.50 (14)	C1—C16—C17—C18	−177.77 (15)
C4—C5—C6—C7	0.7 (2)	C8—C13—C12—C11	−0.7 (2)
C4—C3—C2—C1	−1.3 (2)	C4—C13—C12—C11	178.83 (15)
C4—C3—C2—C14	176.17 (14)	C13—C12—C11—C10	−0.1 (3)
N1—C1—C2—C3	1.3 (2)	C2—C14—C15—O1	60.50 (18)
C16—C1—C2—C3	−177.99 (13)	C17—C16—C21—C20	−1.1 (3)
N1—C1—C2—C14	−176.11 (14)	C1—C16—C21—C20	177.39 (17)
C16—C1—C2—C14	4.6 (2)	C8—C9—C10—C11	0.1 (3)
C12—C13—C8—C9	1.2 (2)	C12—C11—C10—C9	0.4 (3)
C4—C13—C8—C9	−178.32 (13)	C18—C19—C20—C21	0.1 (3)
C12—C13—C8—C7	−178.16 (14)	C22—C19—C20—C21	179.70 (17)
C4—C13—C8—C7	2.3 (2)	C16—C21—C20—C19	0.7 (3)
N1—C1—C16—C17	89.77 (18)		

### Hydrogen-bond geometry (Å, °)

**Table:**

Cg is the centroid of the N1,C1–C5 pyridine ring.

**Table:**

*D*—H···*A*	*D*—H	H···*A*	*D*···*A*	*D*—H···*A*
O1—H1···N1^i^	0.93 (3)	1.98 (3)	2.9110 (18)	174 (2)
C21—H21A···Cg^ii^	0.93	2.97	3.7358 (19)	140.

Symmetry codes: (i) *x*+1, *y*, *z*; (ii) −*x*+1, −*y*+2, −*z*+1.
